# Improvement in the stability and bioavailability of pumpkin lutein using β‐cyclodextrin microcapsules

**DOI:** 10.1002/fsn3.3288

**Published:** 2023-02-28

**Authors:** Zhenxia Shi, Gaoyuan Kong, Fangfang Wang, Hui Gao, Anran Wei, Sizhu Ren, Xunyou Yan

**Affiliations:** ^1^ College of Life Sciences Langfang Normal University Langfang China; ^2^ Technical Innovation Center for Utilization of Edible and Medicinal Fungi in Hebei Province Langfang China; ^3^ Edible and Medicinal Fungi Research and Development Center of Hebei Universities Langfang China; ^4^ Qingdao Agricultural Technology Extension Station Qingdao China

**Keywords:** lutein, microcapsule, ultrasonic‐assisted extraction, β‐cyclodextrin

## Abstract

Growing concerns about food nutrition and food supplies have encouraged the development of effective constituents. Lutein is an important nutrient element, and its health benefits are gradually being recognized. Lutein, as a carotenoid antioxidant, can protect cells and organs from damage caused by free radicals. However, in processing, storage, and usage, lutein is unstable and often undergoes isomerization and oxidative decomposition, which limits its wide range of applications. β‐Cyclodextrin is an ideal substrate to prepare microcapsule structures, which are highly biocompatible and nontoxic. During the lutein encapsulation process, ideal β‐cyclodextrin microcapsules were used to form inclusion compounds. The results reveal that the encapsulation efficiency of the microcapsules reached 53%. Moreover, using ultrasonic‐assisted extraction can easily and efficiently purify lutein. In addition, the capability of the β‐cyclodextrin composite shell can enhance the bioactive molecules' activity and stability.

## Graphical abstract

Lutein‐inclusion ß‐cyclodextrin nanoparticles rationally established in a common solvent. A novel ß‐cyclodextrin microcapsule carrier was synthesized. Lutein encapsulation is an advantageous alternative to increase lutein stability.

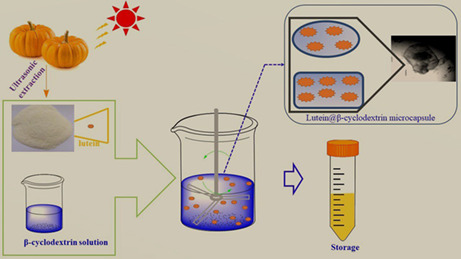

## 1 INTRODUCTION

Lutein is a xanthophyll carotenoid (Long et al., 2019) that has been widely used for its nutraceutical properties (Rezende et al., 2020). Lutein has a C40 isoprenoid backbone with oxygen‐containing rings at both ends, and it gives yellow–orange fruits and vegetables their color (Bhat & Mamatha, 2021). Lutein has significant clinical functions in maintaining the normal physiological function of the human body, and this is currently thought to be mainly due to its role as an antioxidant in the anthropic retina, defending eyes from inflammation and oxidative damage (Kijlstra et al., 2012). Therefore, the effect of lutein's antioxidant properties on the retina is critical to decreasing the speed of the oxidative damage associated with age‐related macular degeneration (Rezende et al., 2020). They could also help in counteracting a decline in pulmonary function (Debora et al., 2016). Lutein is a fat‐soluble compound that is easily deposited and absorbed by many body tissues (Wang et al., 2021). In addition, lutein may have a certain protective action against the oxidation of docosahexanoic acid in the human brain's active sites (Mohn et al., 2017). All these main clinical benefits of disease resistance are mostly due to lutein's ability to effectively resist damage from free radicals to cells and organs (Boehm et al., 2020). However, because the human body cannot synthesize this crucial and bioactive substance on its own, lutein can only be obtained from food, primarily green vegetables, eggs, and some fruits (Crichton et al., 2015). Furthermore, as an important light‐ and heat‐sensitive pigment, lutein is easily inactivated and damaged by heat, oxygen, etc., after its extraction from a raw material, which restricts its utilization efficiency and storage life (Boer et al., 2020; Syamila et al., 2019). To overcome these adverse effects, lutein can often be combined with other substances to achieve improved stability. Microencapsulation, a physiochemical approach to packaging bioactive molecules in a protective material, has been widely used in recent years to overcome the limitations of food ingredients (Li et al., 2021). However, most shell materials are poisonous after decomposition, nonrenewable, and have low biocompatibility. Therefore, looking for a new and environmentally friendly wall material will be the key to whether this technology can be widely used.

β‐Cyclodextrin is regarded as one of the most promising cyclic oligomers of glucose (Zheng et al., 2021) and exhibits availability, nonimmunogenicity, biodegradability, biocompatibility, and low toxicity (Sherje et al., 2017). Its abundant porous structure with a hydrophobic intercavity and a hydrophilic outer surface has attracted the attention of organic substrates for encapsulating natural bioactive molecules, because this structure can prevent natural bioactive molecules from degrading before consumers ingest them (He et al., 2021; Silva et al., 2021). In addition, each glucopyranose ring in each β‐cyclodextrin has a different cavity size, offering infinite possibilities for more diverse biomolecular payloads (Köse et al., 2021). β‐Cyclodextrin comes from a wide range of sources, but currently, it is mainly obtained from starch with enzymatic degradation (Crini et al., 2021). Due to these merits, β‐cyclodextrin has become preferred in numerous studies. Pumpkin is a commonly grown vegetable that is believed to have luxuriant lutein (Seo et al., 2005). Herein, in this study, the extraction conditions of lutein were explored, and the leakage test was evaluated after lutein encapsulation by the β‐cyclodextrin coating. To our knowledge, this is the first study on supersonic wave extraction of lutein in pumpkin and encapsulating lutein with renewable biomaterials to improve its storage stability and prolong its half‐life to solve the instability of pumpkin lutein and low encapsulation efficiency of this fat‐soluble vitamin in monolayer materials.

## 2 MATERIALS AND METHODS

### 2.1 Materials

All solvents used were HPLC or reagent grade. The pumpkin was supplied by a local supermarket in Langfang. The lutein standard (>97% purity) was purchased from Shanxi Kangyue Bio‐Chem Technology Co. Aladdin Bio‐chem Technology Co., Ltd. supplied anhydrous ethanol, β‐cyclodextrin, methanol, sucrose ester, and other medical products.

Pumpkin was sliced first, and then the semimanufactured product was freeze‐dried at −30°C for 36 h in a refrigerator. Afterward, the frozen products were put into a vacuum freeze‐dryer with a cryotrap temperature of −54°C and a vacuum degree of 0.09 Mbar, and the drying time lasted approximately 45 h. After grinding with liquid nitrogen and screening with 100 mesh sieves, the pumpkin powder was prepared and stored at 4°C for further use.

### 2.2 Determination of optical density and standard curve of pumpkin lutein

Lutein made to imitate that found in pumpkin was prepared by dissolving approximately 0.09 mg standard lutein into 2 ml ethyl alcohol, transferring this solution to a 50 ml volumetric flask, and diluting it with deionized water until the flask was full; then, the stock solution was obtained. Then, 1, 2, 3, 4, 5, and 6 ml of stock solution was taken, and distilled water was added until the total volume of liquid was 10 ml. Afterward, different concentrations of standard solution were obtained. Each solvent was prepared fresh before use.

### 2.3 Pumpkin lutein preparation by ultrasonic extraction

Homogeneous freeze‐dried pumpkin powder (1 g) was mixed with an ethanol solution (V/V, 90%). After vortex mixing, the samples were extracted at 20, 30, 40, 50, 60, 70, and 80°C for 30 min. After cooling the supernatant to room temperature, the solution was centrifuged and filtered to remove the impurities. By measuring the wavelength at 445 nm, the effect of temperature was first measured using this method. Similarly, the extraction time (20, 30, 40, 50, 60, 70, 80 min), liquid–solid ratio of ethanol and powder (m/v, 5:1, 10:1, 15:1, 20:1, 25:1, 30:1 g/ml), ethanol concentration (V/V, 40%, 50%, 60%, 70%, 80%, 90%, 100%), and ultrasonic frequency (160, 200, 240, 320, 360, 400 w) were all determined. At each predetermined extraction condition, the samples were treated with ultrasound and those without ultrasound, which were marked as ULT and COT, respectively. Finally, the optimum extraction conditions were confirmed. Each sample was repeated three times to measure the completeness of the pumpkin lutein extraction method.

### 2.4 Confirmation of optimum synthesis conditions by orthogonal experiment

To further determine the optimal extraction conditions, orthogonal experiments with four factors and three levels (L_9_ (3^4^)) were designed. Not only the temperature and its response time but also the ratio (coefficient proportion of ethanol and powder) and alcoholic concentration were considered. By using range analysis, the final selective factor and its level were finally confirmed (Table 1). The design is as follows:

**TABLE 1:** Factors and levels of orthogonal experiment.

Level	Temperature (°C)	Time (min)	Ratio	Ethanol concentration (%)
1	50	30	10	80
2	60	40	15	90
3	70	50	20	100

### 2.5 Preparation of lutein microcapsules

A certain amount of sucrose ester was added to the concentrated solution of lutein to fully emulsify lutein, and the ratio of lutein in the supernatant to the mass of sucrose ester was approximately 10:1. Then, β‐cyclodextrin was added to an appropriate amount of distilled water at 60°C and stirred to dissolve so that the β‐cyclodextrin could fully swell and disperse, and a saturated solution of carriers was obtained, which should be continued to cool to approximately 50°C for incubation. Afterward, the emulsified solution was stirred and added to the solution of the carrier. Ultrasound was carried out for 40 min at 35°C with an ultrasonic power of 40%. Finally, the obtained precipitate (lutein microcapsules) was washed with deionized water several times and lyophilized for further use at 4°C.

### 2.6 Encapsulation efficiency

The prepared lutein microcapsules with a certain quality were mixed with water. After the outer microcapsules were destroyed mechanically, lutein was extracted by adding organic solvent (ratio of methanol to ethanol was 1:1) until the mixed solution was colorless. When lutein was completely transferred to the organic phase, the content of lutein embedded in the microcapsules was determined. Afterward, the stock solution of pumpkin lutein used to prepare these microcapsules was measured again to evaluate the encapsulation efficiency.

$$A=\frac{m_{1}}{m_{2}}\times 100\%$$

*A* is the encapsulation efficiency, *m*_1_ is the amount of lutein extracted after crushing microcapsules, and *m*_2_ is the content of lutein in the original liquid.

### 2.7 Performance evaluation of lutein microcapsules

#### 2.7.1 Morphological observation

The morphology and sizes of the microcapsules were characterized by using three‐dimensional video microscopy (OLYMPUS‐CX23). The size of the microcapsules was determined in optical micrographs (magnification 40×) with analysis software (Image View).

#### 2.7.2 Water content

The petri dish was predried and weighed as *m*_0_, and then the weight of lutein microcapsules was weighed as *m*_1_, and after this, the lutein was baked in an oven at 105°C until the weight was constant. Afterward, the weight was recorded as *m*_2_. The water content was calculated according to the following formula.

$$B=\frac{m_{2}-m_{0}}{m_{1}-m_{0}}\times 100\%$$

where *B* is the water content, *m*_1_ is the weight of the lutein microcapsules before drying, and *m*_2_ is the weight of the dried product.

#### 2.7.3 Stability

Approximately 0.100 g of microcapsule pumpkin lutein and 1 ml of a concentrated pumpkin lutein stock solution (containing approximately 0.03 g of lutein) were taken. The microcapsule lutein was dissolved in 1 ml of anhydrous ethanol, and then distilled water was added to 100 ml for the temperature stability test. Twenty milliliters of the two samples were heated at 20, 40, 60, 80, and 100°C for 30 min. Finally, the changes in the OD value of microcapsule pumpkin lutein were detected to determine the stability.

The operation steps were similar, and 20 ml of the two solutions were taken, and pH values were adjusted to 2, 4, 6, 8, and 10 by HCl and NaOH to detect the change in OD value of microcapsule pumpkin lutein to detect the reaction stability.

For light stability testing, natural light exposure at room temperature (25°C) was required for 2, 4, 6, 8, and 10 days, with distilled water as the control, and absorbance was measured at 445 nm.

### 2.8 Data analyses

SPSS Statistic V21.0 was used, and data were processed by significance analysis (mean, standard deviation).

## 3 RESULTS AND DISCUSSION

### 3.1 Confirmation of optical density and the standard equation

Raw optical density readings were exported from the spectrophotometer and processed in Excel. The absorption spectra of pumpkin lutein are shown in Figure S1, which fully displays the relationship between the OD value and its corresponding wavelength. The maximum absorption peak occurred at 445 nm between 400 and 500 nm; therefore, we chose 445 nm as the measuring wavelength in subsequent studies.

A standard curve was derived from the serial dilutions in a customary way. Relative concentrations were expressed in arbitrary units. Plotting was performed with concentration as abscissa and OD value as an ordinate. The least‐square fit was used as the standard curve, and the standard equation was obtained: y=2.3571x+0.0214.

### 3.2 Single factor of extraction conditions

**FIGURE 1:**
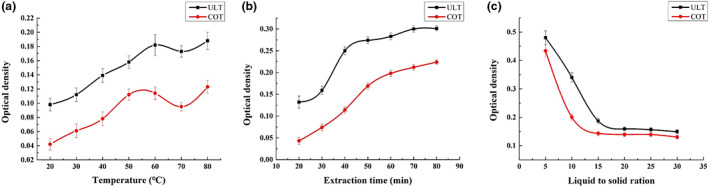
Relationship between temperature/extraction time/liquid to solid ratio and lutein extraction yield. (a) Temperature; (b) extraction time; (c) liquid to solid ratio.

The relationship between temperature and lutein extraction efficiency is shown in Figure 1a. As the temperature increased, the absorbance gradually increased. When the temperature exceeded 60°C, the extraction efficiency of OD gradually showed a downward trend, which is most likely because the structure of lutein is damaged at high temperatures (Xiao et al., 2018). However, when the temperature reached 80°C, the absorbance value suddenly increased, which may be due to the concentration of the extract caused by the increase in ethanol volatilization in the solution (Cunningham et al., 2018). Therefore, 60°C was selected as the optimal extraction temperature for this pigment.

With the extension of extraction time, the extraction efficiency of lutein between different methods also represented an increasing trend (Figure 1b). Sonication is an effective tool for extracting organics, it requires simple equipment, and liquid–solid separations can usually be achieved in a short time (Sunardi & Nisa, 2021). Compared with the two methods, microwave‐assisted extraction was time‐saving and more efficient, which has a certain auxiliary effect on lutein extraction in terms of extraction time. From the final OD differences, time savings and high efficiency are undeniable in ultrasonic treatment. However, the ultrasonic extraction of lutein slowed growth after 40 min, while the water bath extraction after 60 min still increased, indicating that ultrasonic extraction can shorten the equilibrium reaction and improve the extraction rate of lutein.

As shown in Figure 1c, the OD value of the two treatments showed an increasing trend in the process of the solid–liquid ratio increasing, which indicated that lutein was in full contact with ethanol in the beginning and had been completely exact. When in a low solid to liquid ratio, the solvent plays an essential dilution role, and when the proportion of pumpkin lutein was low, this resulted in a small OD value.

As the proportion of solids increases gradually, the OD value begins to increase. Meanwhile, for general water bath extraction, the OD value began to change significantly when the solid–liquid ratio reached 10:1. Comparatively speaking, the OD value began to change significantly when the solid–liquid ratio reached 15:1 for ultrasonic‐assisted extraction, showing that the dilution effect of the solvent is no longer dominant.

In contrast, the OD of the ultrasonic treatment continued to increase with increasing solid–liquid ratio, indicating that the contact between the pigment and ethanol was relatively sufficient at the beginning, and it could dissolve completely. Additionally, for the water bath extraction, the OD began to change when the solid–liquid ratio reached 1:10, and for the ultrasonic‐assisted extraction, the solid–liquid ratio began to change significantly and reached 1:15, which further showed that ultrasonication plays an important role in reducing production costs and saving raw materials.

**FIGURE 2:**
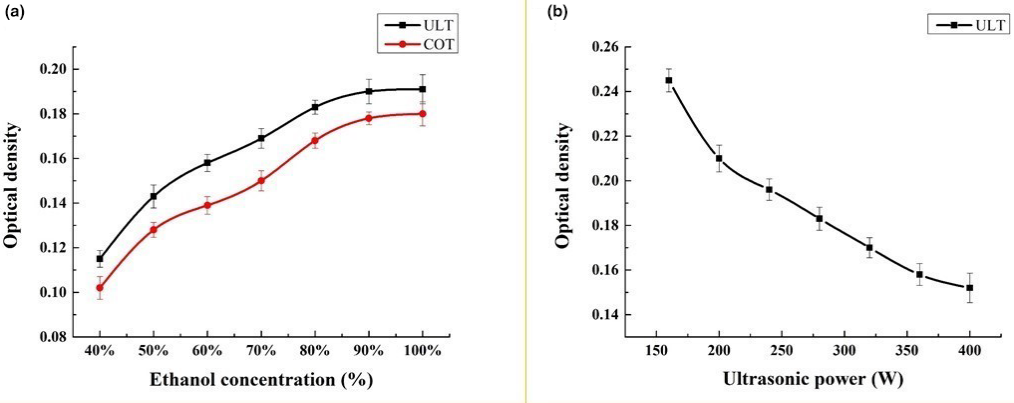
Relationship between different ethanol concentrations/ultrasonic powers and lutein extraction yields. (a) Different ethanol concentration and its corresponding OD value; (b) Different ultrasonic power treatment and its corresponding OD value.

Ethanol is a polar solvent often used in extraction and separation (Xie et al., 2019). Therefore, exploration of the concentration is necessary for optimization of the whole process. As shown in Figure 2, both ultrasonic treatment and water bath extraction showed an increasing trend in the extraction efficiency of lutein with increasing ethanol concentration. When the concentration of ethanol reached 100%, the extraction rate reached its maximum value, and there was no significant difference between the two treatments in the extraction rate of ethanol (Figure 2a).

The cavitation and crushing effect produced by ultrasonic treatment can effectively improve cell crushing efficiency and accelerate the diffusion release of its contents and solubility in the solvent, but the strong mechanical vibration of ultrasonication may destroy the material structure and then the physiological activity and yield of lutein and proteins (Bussemaker & Zhang, 2013; Zhang et al., 2011). The highest OD value occurred when the ultrasonic power was 40% (150 W) of the total power of 400 W (Figure 2b), which means lutein has the best extraction effect at this power. This is probably because although certain ultrasonic treatments can increase the cell fragmentation effect and increase the amount of lutein dissolution in a certain range, higher ultrasonic power will destroy lutein and lead to a low extraction efficiency.

### 3.3 Orthogonal experiment and final extraction conditions

The orthogonal test results and variance analysis results are shown in Tables 2 and 3, respectively. Regarding the effect of various factors for extracting pumpkin lutein, temperature was the most important factor, and ethanol concentration was the least important factor. Therefore, A2B3C1D3 was selected for the extraction, which means that the maximum pigment extraction efficiency could be obtained using ethyl alcohol with a liquid–solid ratio of 10 and an extraction time of 50 min at 60°C.

**TABLE 2:** Trials and results of orthogonal experiment design.

Number	Temperature/°C (A)	Time/min (B)	Liquid–solid/ml:g (C)	Ethanol concentration/% (D)	OD	Concentration, μg/ml
1	50	30	10	80	0.18	60.2471
2	50	40	15	90	0.157	50.4474
3	50	50	20	100	0.137	41.9259
4	60	30	15	100	0.163	53.0038
5	60	40	20	80	0.162	52.5778
6	60	50	10	90	0.274	100.2983
7	70	30	20	90	0.098	25.3089
8	70	40	10	100	0.247	88.7942
9	70	50	15	80	0.169	55.5603
K_1j_	152.6204	138.5598	249.3396	168.3852		
K_2j_	205.8799	191.8194	159.0115	176.0546		
K_3j_	169.6634	197.7845	119.8126	183.7239		
K_4j_	50.8735	46.1866	83.1132	56.1284		
K_5j_	68.6252	63.9398	53.0038	58.6849		
K_6j_	56.5545	65.9282	39.9375	61.2413		
R_j_	17.7517	19.7416	43.1757	5.1129		

**TABLE 3:** Analysis of variance of the regression parameters.

Sources of variation	Degree of freedom (df)	Sum of squares (SS)	Mean sum of square (MS)	*F*	Fa
A	2	493.1859	246.593	12.5772	*F*_0.05(2,2)_ = 19 *F*_0.01(2,2)_ = 99
B	2	708.8592	354.4296	18.0772	
C	2	2941.4403	1470.7202	75.0123	
Error (D)	2	39.2127	19.6064	1	
Total variation	8	4182.6981	—	—	—

*Note*: The results of variance analysis illustrate that the C factor has a significant effect on pumpkin lutein extraction yield.

#### 3.3.1 Preparation and embedding rate of microcapsules

**FIGURE 3:**
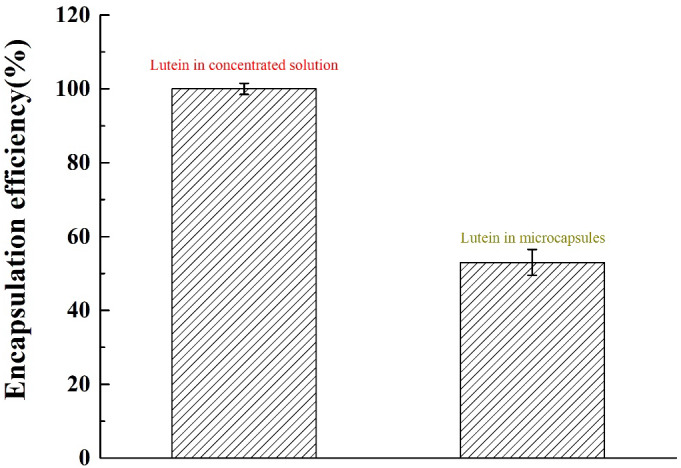
Total lutein content in concentrated solution and content of lutein in microcapsules.

After emulsified β‐cyclodextrin monomer polymerization, a small cyst will form to wrap the lutein. The efficiency of encapsulation was used to assess the impact of preparing reacting lutein products. If lutein was coated by the microcapsules, good stability and good storage would be achieved. However, a lower encapsulation rate will directly result in lutein being easily damaged by high temperatures, oxygen, and acidic environments during processing. β‐Cyclodextrin was used as the carrier, and the embedding rate reached 53% (Figure 3), indicating that more than 50% of lutein was protected by the carrier during microcapsule preparation. However, the inclusion rate is difficult to increase, which is most likely due to the reduction of the solubility of β‐cyclodextrin by the ethanol contained in the concentrate and the incomplete solubility of cyclodextrin due to the difference in solubility of cyclodextrin at different temperatures.

#### 3.3.2 Micromorphology of lutein microcapsules

**FIGURE 4:**
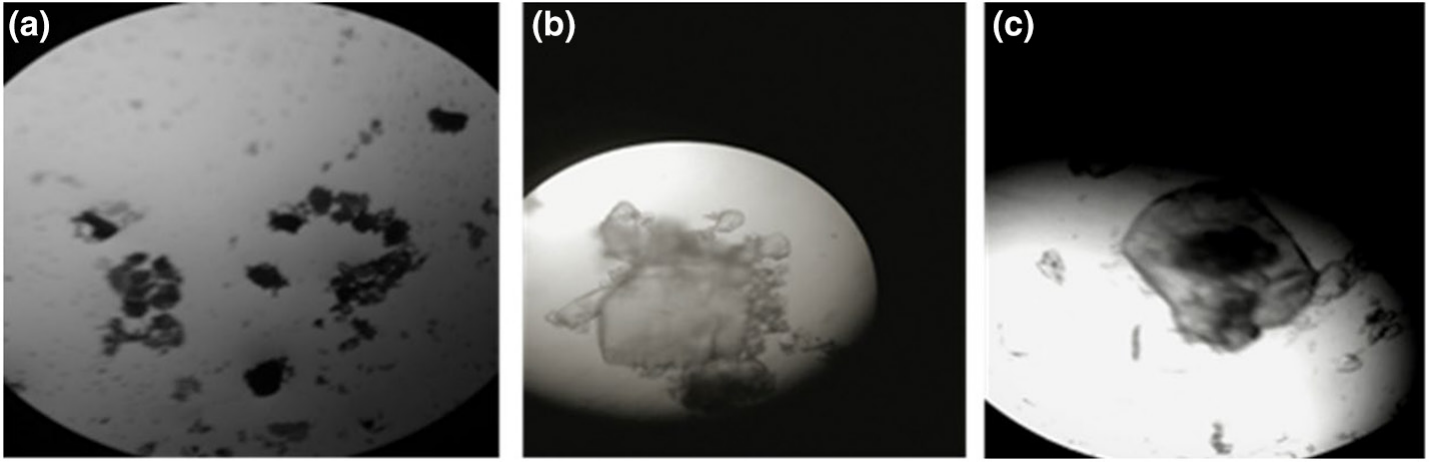
Micromorphology of all products. (a) Lutein; (b) β‐cyclodextrin; (c) Lutein microcapsules.

**FIGURE 5:**
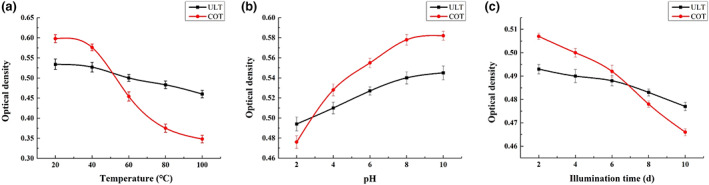
Effects of different external factors on the stability of lutein in a natural setting and microcapsules. (a) Temperature; (b) pH; (c) Illumination time.

Figure 4 shows the micromorphologies of all products at 400 times resolution. Counterintuitively, the lutein microcapsules showed an approximately elliptic shape with a diameter of 20–150 nm, which was consistent with previous research (Martínez‐Pérez et al., 2012). The difference in particle size may be related to the dissolution of β‐cyclodextrin during the embedding process. The presence of a black core in Figure 5c confirms that lutein was successfully packaged in a β‐cyclodextrin coating.

#### 3.3.3 Stability evaluation

Lutein was packaged in β‐cyclodextrin microcapsules, and its stability was measured, including water content, temperature, pH, and light. The high water content will lead to the proliferation of microorganisms and degradation of the active ingredients in food, thus reducing the shelf life. After drying and weighing to a constant weight, the moisture content was approximately 5.98%, which facilitates the long‐term preservation of pumpkin lutein microcapsules.

Increasing temperature directly leads to a decrease in lutein OD, which indicates that high temperature has a certain destructive effect on lutein (Figure 5a). However, overall, the absorbance of microcapsule lutein changed slightly. When heated to 80°C, the absorbance of native lutein decreased to 36.2%, while the absorbance of microencapsulated pumpkin lutein only decreased to 9.5%. At 100°C, the absorbance of native lutein and lutein in the microcapsule decreased by approximately 40.8% and 13.8%, respectively. Thus, β‐cyclodextrin microcapsules play an important role in protecting lutein against high‐temperature damage. The physical stability of the lutein solution was significantly affected by pH (Davidov‐Pardo et al., 2016). The absorbance changes of microcapsule lutein and lutein under different pH values are shown in Figure 5b. The more the pH value decreased, the more the absorbance value showed a certain positive correlation and continuously decreased. When pH reached 2, the absorbance decreased by approximately 18.2%. However, the absorbance of lutein within microcapsules showed a certain rigidity because when the pH value dropped to 2, the absorbance decreased by only 9.4%. Pumpkin lutein was very unstable in highly acidic environments, while lutein within microcapsules could be used in a wider range of acidic and basic environments.

Pumpkin lutein is more sensitive to external light (Silva et al., 2017). With the extension of illumination time, the absorbance decreases (Figure 5c). After irradiation for 10 days, the absorbance decreased by 8.1%, while under the same conditions, the microcapsule pumpkin lutein showed a relatively stable performance, with an absorbance decrease of only 3.2%. Therefore, under light conditions, lutein inclusion microcapsules are more suitable for food production and storage.

## 4 CONCLUSIONS

In summary, the purification and optimization of the process by ultrasonic extraction is beneficial to the extraction of lutein and the maintenance of its biological activity. In addition, a new and environmentally friendly β‐cyclodextrin microcapsule pumpkin lutein was rationally prepared and designed. The β‐cyclodextrin microcapsule can ensure that lutein maintains a more stable state under acidic, light, and other adverse conditions. The present study has shown that microencapsulated lutein could be a promising candidate for the food industry and processing industry.

## FUNDING INFORMATION

This work was supported by Scientific Research Foundation of Langfang Normal University (grant number XBQ202028).

## CONFLICT OF INTEREST STATEMENT

The authors declare that they have no known competing financial interests or personal relationships that could have appeared to influence the work reported in this paper.

## Supporting information


Figure S1

## Data Availability

The data that support the findings of this study are available in the supplementary material of this article.
