# Supplementary material for: Improvement in the stability and bioavailability of pumpkin lutein using β‐cyclodextrin microcapsules
Source: Food Sci Nutr. 2023 Feb 28;11(6):3067–74. doi: 10.1002/fsn3.3288 (PMC10261736; doi:10.1002/fsn3.3288)
Supplement: Supplementary file 1 — Figure S1 [file FSN3-11-3067-s001.docx]

**Supplementary data**

**Fig.S1**


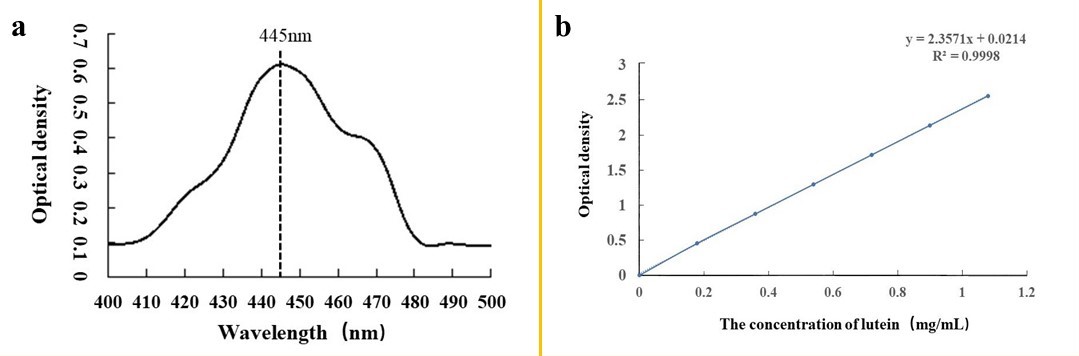


Fig.1 Absorption spectrum of pumpkin lutein and its standard curves. a. Full wavelength scanning of pumpkin lutein；b. Standard curves and regression equations;
